# A Computational Approach with Biological Evaluation: Combinatorial Treatment of Curcumin and Exemestane Synergistically Regulates DDX3 Expression in Cancer Cell Lines

**DOI:** 10.3390/biom10060857

**Published:** 2020-06-04

**Authors:** Shailima Rampogu, Seong Min Kim, Minky Son, Ayoung Baek, Chanin Park, Gihwan Lee, Yumi Kim, Gon Sup Kim, Ju Hyun Kim, Keun Woo Lee

**Affiliations:** 1 Division of Life Sciences, Division of Applied Life Science (BK21 Plus), Plant Molecular Biology and Biotechnology Research Center (PMBBRC), Research Institute of Natural Science (RINS), Gyeongsang National University (GNU), 501 Jinju-daero, Jinju 52828, Korea; shailima.rampogu@gmail.com (S.R.); minky0710@gmail.com (M.S.); wydkwydk86@gmail.com (A.B.); chaninpark0806@gmail.com (C.P.); pika0131@naver.com (G.L.); miya.candy@gmail.com (Y.K.); 2 Research Institute of Life Science and College of Veterinary Medicine, Gyeongsang National University, 501 Jinju-daero, Jinju 52828, Korea; ksm4234@naver.com (S.M.K.); gonskim@gnu.ac.kr (G.S.K.); 3 Department of Chemistry (BK 21 plus), Research Institute of Natural Science (RINS), Gyeongsang National University, Jinju, Gyeongnam 52828, Korea; juhyun@gnu.ac.kr

**Keywords:** DDX3, cancers, natural compounds, combinatorial treatment

## Abstract

DDX3 belongs to RNA helicase family that demonstrates oncogenic properties and has gained wider attention due to its role in cancer progression, proliferation and transformation. Mounting reports have evidenced the role of DDX3 in cancers making it a promising target to abrogate DDX3 triggered cancers. Dual pharmacophore models were generated and were subsequently validated. They were used as 3D queries to screen the InterBioScreen database, resulting in the selection of curcumin that was escalated to molecular dynamics simulation studies. In vitro anti-cancer analysis was conducted on three cell lines such as MCF-7, MDA-MB-231 and HeLa, which were evaluated along with exemestane. Curcumin was docked into the active site of the protein target (PDB code 2I4I) to estimate the binding affinity. The compound has interacted with two key residues and has displayed stable molecular dynamics simulation results. In vitro analysis has demonstrated that both the candidate compounds have reduced the expression of DDX3 in three cell lines. However, upon combinatorial treatment of curcumin (10 and 20 μM) and exemestane (50 μM) a synergism was exhibited, strikingly downregulating the DDX3 expression and has enhanced apoptosis in three cell lines. The obtained results illuminate the use of curcumin as an alternative DDX3 inhibitor and can serve as a chemical scaffold to design new small molecules.

## 1. Introduction

DEAD box protein 3, DDX3, also referred to as DDX3X, belongs to the family of DEAD box RNA helicase [1]. These RNA helicases demonstrate a specific motif called the DEAD/H (Asp-Glu-Ala-Asp/His) [2]. Although, exploration for the key functions of human DEAD/H box proteins is still underway, it has been evidenced that these proteins are involved with energy-dependent RNA metabolism, such as ribosome biogenesis, translation, pre-mRNA splicing, RNA editing, and RNA turnover [2,3,4]. Of all the members of DEAD-box proteins, DDX3 is highly conserved. In humans, there exists at least two pseudo genes and two DDX3 homologs, DDX3X and DDX3Y, sharing about 92% of protein sequence identity with varied functional and expression patters across various organs [2]. Notably, the protein is known to transport between cytoplasm and nucleus [1,5], however is primarily localized in cytoplasm in a majority of human tissues and cell lines [1]. DDX3 is positioned on the X-chromosome bands p11.3 --> p11.23 [2,6] whose deletion is embryonically lethal. The function of DDX3 is widely explored in several viruses as an important factor for replication [7] and its role in cancer is a recent advancement [2]. This protein is evidenced to manifest anti-apoptotic properties, invasion, is associated with migration and its elevated levels in mammary epithelium triggers breast tumorigenesis. [8,9,10,11,12]. Additionally, DDX3 is connected with cell cycle progression [13] and inhibiting DDX3 brings about the G1-arrest [14].

Structurally, DDX3 is made up of two recA-like domains and 12 conserved motifs. The crystal structure of DDX3 has been co-crystallized with adenosine monophosphate (AMP) [2,15,16] existing between the Q motif and the P-loop. The adenine moiety of the AMP interacts with the residues prompted from the Q motif such as Arg202 and Gln207 while the phosphate group interacts with the residues originating from the P-loop, Gly227, Ser228, Gly229, Lys230 and Thr231. Additionally, the purine interacts with the phenyl group of Tyr200 holding the AMP firmly at the nucleotide binding pocket. Due to the flexible nature of the P-loop, the DDX3 may demonstrate several conformations, while the DDX3 with AMP binding illustrates an open conformation [2]. Correspondingly, targeting the nucleotide-binding pocket of DDX3 by small molecules might be an effective strategy in reducing the cancer cases.

Of late, several inhibitors that target the DDX3 have been identified, one of which is RK-33 [17], specifically designed to occupy the ATP-binding pocket of DDX3. Furthermore, this inhibitor demonstrated selective inhibition towards DDX3 over other proteins of the same family and hinders the RNA helicase activity [11,14]. Moreover, RK-33 has demonstrated selective anticancer effect in Ewing sarcoma [18] and acts as a radiosensitizer [14,19]. Another inhibitor, ketorolac salt is evidenced to inhibit DDX3 and proved as potent candidate compound in treating oral cancer [20]. It is documented that the compound NZ51, which is a ring expanded nucleoside analogue has hindered the motility and viability of breast cancer cells, particularly targeting the DDX3 [21]. Encouraged by these reports, we intended to perform molecular modelling and virtual screening techniques to retrieve potential candidate compound and evaluate its efficacy on three cell lines, the MCF-7, MDA-MB-231 (breast cancer) and HeLa (cervical cancer).

Notably, DDX3 is overexpressed in breast cancer [22], colorectal cancer [23,24], liver cancer [2], lung cancer [2,14,19] and oral cancer [2,20]. Breast cancer is one of the predominantly noticed cancers in women associated with 570,000 deaths in the year 2015 [25]. DDX3 is reported to have been in elevated levels in metastatic breast cancer particularly, in triple negative cancer cases [22]. Additionally, a positive correlation was determined between overexpression of hypoxia-inducible factor 1-alpha (HIF-1α) and DDX3 expression in breast cancer [26]. Cervical cancer is one of the major cancer deaths observed in women and is ranked fourth among female cancers [27]. Therefore, in the current investigation, we aimed at finding a natural compound as an inhibitor for DDX3 in three cell lines.

## 2. Materials and Methods

### 2.1. Pharmacophore Generation

The generation of the pharmacophore model is a crucial event as it is employed to retrieve the potential compounds from a given database. For the current investigation, two different pharmacophore models have been utilized namely, the common feature pharmacophore model generation and the receptor-based pharmacophore model generation. In the common feature pharmacophore model generation, four reported inhibitors with an IC_50_ value lower than 10,000 nM were chosen, while the receptor based pharmacophore model was generated employing the protein with the PDB code 2I4I [15] and are detailed below.

### 2.2. Common Feature Pharmacophore Generation

**Figure 1:**
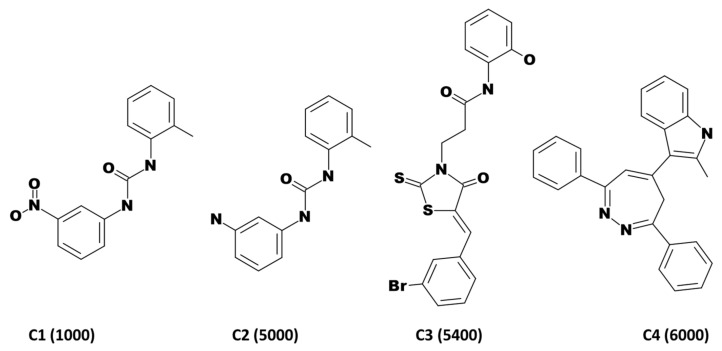
2D structures of compounds employed for common feature pharmacophore generation. The IC_50_ values in nM are represented in parenthesis.

From a set of known ligands obtained from the binding db [28], a pharmacophore was generated employing the *Common Feature Pharmacophore Generation*. This utilizes *HipHop* algorithm from a set of given active ligands to produce a common feature pharmacophore [29]. For the current investigation, the interfeature distance was taken as 2.00 with maximum pharmacophores as 10, while retaining all the other parameters as default. The features chosen for the pharmacophore generation are hydrogen bond acceptor (HBA), hydrogen bond donor (HBD), hydrophobic (HYP), hydrophobic aromatic (HA) and ring aromatic (RA), respectively. The most active known compounds were retrieved from the binding db [28,30,31] to extract the key features for biological activity as demonstrated in Figure 1. Hereinafter the generated pharmacophore is referred to as pharm1.

### 2.3. Receptor Based Pharmacophore Generation

Also known as structure-based pharmacophore modelling, this method uses the structure of a protein in complex with its co-crystallized ligand to generate selective pharmacophore models exploiting the receptor ligand interactions [32]. Correspondingly, the *Receptor-Ligand Pharmacophore Generation* protocol was enabled with maximum pharmacophores as 10 with minimum and maximum features as 4 and 5, respectively, while retaining the default settings of all the other parameters. The protein for the current study was downloaded from the protein data bank (PDB code 2I4I), co-crystallized with adenosine monophosphate. Hereinafter the generated pharmacophore model is labelled as pharm2.

### 2.4. Validation of the Pharmacophore Models

Validation of the generated pharmacophore models is a step that involves the evaluation of the models in retrieving the prospective active compounds when subjected to screen larger databases. Accordingly, the pharm1 and pharm2 were judged for their propensity towards the active compounds employing the receiver operating characteristic (ROC) curve. This prediction was conducted alongside the pharmacophore generation. For effective execution of the protocol, a set of four ligands as mentioned in Figure 1 were considered active compounds, while a set of eight compounds derived from binding db were labelled as inactive compounds as represented in Table 1. Subsequently, the area under the curve (AUC) was computed to grade the pharmacophore quality.

### 2.5. Drug-Like Database Formulation from InterBioScreen Database

The validated pharmacophore models were escalated to screen and thereby retrieve the prospective drug-like compounds. For the current investigation, the InterBioScreen database was employed consisting of 59,619 compounds with manual inclusion of known compounds. These compounds were initially explored for their pharmacokinetic and pharmacodynamic properties employing the *ADMET Descriptors* accessible with the DS v18. ADMET stands for Absorption, Distribution, Metabolism, Excretion, and Toxicity and is an important parameter that can serve to promote a drug during developmental process and the upper limit of the values were set as described previously [33]. Accordingly, the absorption level was fixed at 0 and 1, the blood brain barrier (BBB) was opted as 2 and 3 and the solubility was secured at 3 and 4. The filtered compounds were upgraded to estimate their oral bioavailability and thus can be labelled as drug-like compounds. This was achieved by enabling the *Filter by Lipinski* obtainable with the DS [34]. The resultant compounds were upgraded for virtual screening using the two pharmacophore models after enabling the *Ligand Pharmacophore Mapping* in DS.

### 2.6. Virtual Screening of InterBioScreen Database Using Pharm1 and Pharm2

The obtained drug-like compounds were examined for possessing the key features by mapping them, using pharm1 and pharm2 as the 3D queries. From the secured compounds, visual inspection was conducted to select the compounds that mapped with both the models, a criteria adapted which illuminates the potentiality of the compounds. The obtained compounds were upgraded for molecular docking studies to estimate the binding affinities with the protein.

### 2.7. Molecular Docking Studies

Molecular docking studies logically elucidates on the binding affinities between the protein and the ligands, thereby predicting the possible binding modes for the Hit compound. For the current investigation, the CDOCKER programme [35] accessible with the DS was utilized, that operates on CHARMm-based molecular dynamics. From the initial ligand conformation, random conformations were generated using high temperature MD that are correspondingly moved to the binding site. The generation of the candidate poses is achieved by rigid-body rotations and simulated annealing coupled by minimization to refine the ligand pose. To accurately predict the binding mode of the ligand, a total of 100 conformation were generated. The best pose was chosen based upon the highest -CDOCKER interaction energy for the compound from the largest cluster that displayed hydrogen bond interactions with the key residues.

The target for the current study is human DEAD-box RNA helicase (DDX3X) bearing the PDB code 2I4I, in complex with adenosine monophosphate (AMP) [15]. The key residues were marked for all the residues that lie in 10 Å around the AMP. The protein was prepared by removing the heteroatoms and water molecules, while supplementing with the hydrogen atoms and filling the gaps. Upon enabling the *Clean Protein* tool available with the DS, prompts if any gaps are present and are subsequently filled using the *Insert Loop* option and thereafter refined by *Loop Refinement* protocol available with the DS. The prepared protein and the ligands were promoted to molecular docking mechanism to delineate on their binding affinities.

### 2.8. Molecular Dynamics Simulation Studies

Molecular dynamics simulations (MDS) provide knowledge about the dynamic nature of small molecules with the protein counterpart at the atomistic level. The initial structures for the MDS were the protein-ligand complexes that were obtained from the molecular docking studies. In order to accomplish this study, the GROMACS v2016.6 package [36,37] was used, retrieving the protein topologies from CHARMm 27 all-atom force field. The ligand topologies were extracted from SwissParam [38]. The dodecahedron water box was generated and solvated with TIP3P water model to accomplish the simulations followed by the addition of counter ions. The initial structures were relaxed through steepest descent algorithm and energy minimized. Following this, a double step equilibration was conducted, first with constant number of particles, volume, and temperature (NVT) ensemble for 1 ns at 300 K using V-rescale thermostat. Later, the constant number of particles, pressure, and temperature (NPT) equilibration was executed for 1 ns monitoring the pressure of the system at 1 bar using Parrinello-Rahman barostat. The NPT equilibrated structures were promoted to MDS for 50 ns, while retaining the other parameters as default. The results were processed and examined utilizing visual molecular dynamics (VMD) [39], UCSF Chimera [40], DS and GROMACS.

### 2.9. In Vitro Bioassay Validation

#### 2.9.1. Procurement of Cell Lines and Culture

Human MCF-7, MDA-MB-231 and HeLa cell lines were purchased from the Korean Cell Line Bank (KCLB, Seoul, Korea). The MCF-7 and MDA-MB-231 cell lines were maintained in RPMI-1640 medium (Gibco, Life Technologies, Carlsbad, CA, USA) and HeLa cell line were cultured in Dulbecco’s modified Eagle’s medium (DMEM; Gibco) containing 10% (v/v) fetal bovine serum (FBS, Gibco) and 1% penicillin–streptomycin (Gibco) at 37 °C in a humidified atmosphere of 5% CO_2_.

#### 2.9.2. Procurement of Compounds and Reagent

Curcumin and exemestane (Sigma-Aldrich, St. Louis, MO, USA) were diluted in dimethyl sulfoxide (DMSO, Sigma-Aldrich). The DDX3, Bcl-xL primary antibodies was obtained from Cell Signaling Technology (Danvers, MA, USA).

### 2.10. Cell Viability Assay

Cells were cultured in 48 well, plated at a density of 5 × 10^4^ cells per well. The seeded cells were treated with various concentration for 24 h. After incubation, the cells were added with 55 µL of 5 mg/mL 3-(4, 5-Dimethylthiazol-2-yl)-2, 5-diphenyltetrazolium bromide (MTT; Duchefa Biochemie, Haarlem, The Netherlands) solution for 2 h, and the medium was removed which was followed by lysis with DMSO. The absorbance at 570 nm was measured with PowerWave HT microplate spectrophotometer (BioTek, Winooski, VT, USA).

### 2.11. Western Blot Analysis

After treatment, the cells were lysed with RIPA buffer (50 mM Tris-HCl pH 7.5, 0.1% SDS, 1% Triton X-100, 150 mM NaCl, 0.5% Sodium deoxycholate and 2 mM EDTA) containing protease/ phosphatase inhibitor cocktail (Thermo Fisher Scientific, Waltham, MA, USA) at 4 °C for 1 h. The supernatants were collected and quantified using BCA protein assay kit (Thermo Fisher Scientific) according to the manufacturer’s instructions. Protein of 10–20 µg concentration was loaded on 8–12% sodium dodecyl sulfate polyacrylamide gel electrophoresis (SDS-PAGE), and then transferred to polyvinylidene difluoride membrane (PVDF, ATTO, Tokyo, Japan). The membranes were blocked with TBS-T buffer (Tris-buffered saline containing 0.1% Tween 20) containing 5% (w/v) skim milk power for 1 h at 25 °C, and then incubated with primary antibodies for 16 h at 4 °C. After incubation with the secondary antibody for 3 h at 25 °C, the membranes was detected using Clarity™ Western ECL Blotting Substrates (Bio-Rad, Hercules, CA, USA). The quantification of protein expression was analyzed with Image J (Version 1.50i) software (National Institutes of Health, Bethesda, MD, USA).

### 2.12. Statistical Analysis

The data were expressed as mean ± standard error of mean (SEM) and analyzed by GraphPad Prism Version 4.0b (GraphPad Software Inc., La Jolla, CA, USA), for statistical significance using one-way analysis of variance (ANOVA). *p* < 0.05 was considered as statistically significant. All experiments were performed in triplicates.

## 3. Results

### 3.1. Pharmacophore Generation

#### 3.1.1. Common Feature Pharmacophore Generation

Utilizing the four compounds mentioned in Figure 1, a common feature pharmacophore was generated. Initially, the *Feature Mapping* module available with the DS enabled to observe and discern on the key features imbibed by the compounds. Accordingly, the hydrogen bond acceptor (HBA), hydrogen bond donor (HBD), hydrophobic (HYP), hydrophobic aromatic (HA) and ring aromatic (RA) were chosen. The *Common Feature Pharmacophore Generation* protocol has prompted ten pharmacophore models with different features and characters as tabulated in Table 2.

**Figure 2:**
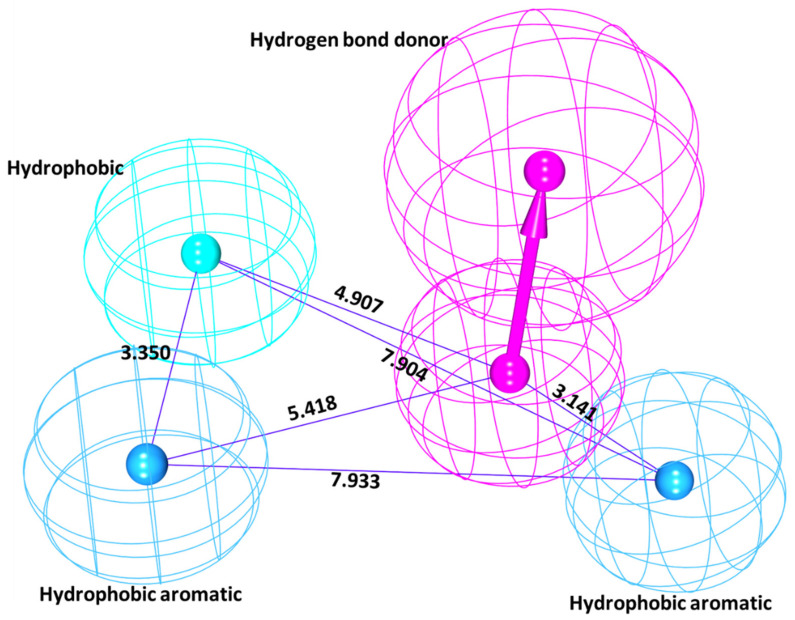
Common feature pharmacophore model and its geometry. Pharma1 has represented four features.

From this the pharmacophore model 1 (pharm1) was chosen based upon the rank and the maximum fit. This resulted in a four featured pharmacophore possessing two hydrophobic aromatic feature, one hydrophobic feature and one hydrogen bond donor feature as depicted in Figure 2.

#### 3.1.2. Receptor-Based Pharmacophore Generation

Receptor-based pharmacophore generation wisely exploits the interactions that exists between the co-crystallized ligand and the residues contributed from the protein. Accordingly, the *Receptor-Ligand Pharmacophore Generation* protocol has generated ten pharmacophore features as represented in Table 3.

**Figure 3:**
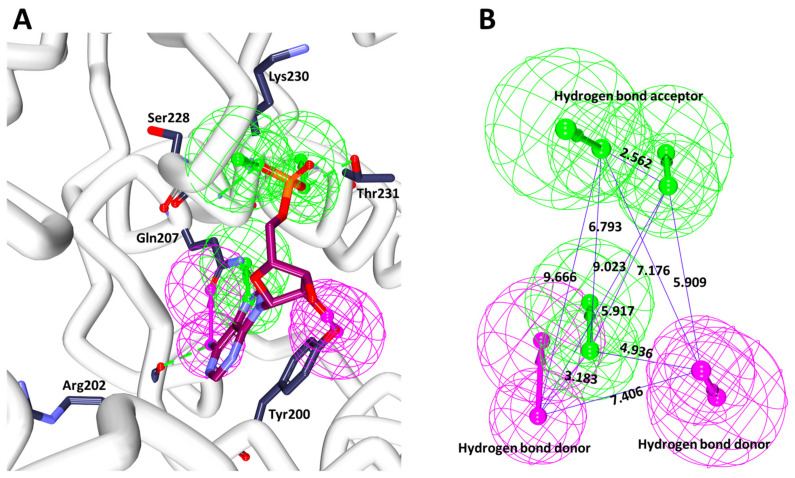
Receptor based pharmacophore model and its geometry. (**A**) Illustrates the features complimentary with the key residues. (**B**) Depicts the interfeature distance.

Interestingly, the ten models have demonstrated the same selectivity score of 9.9146, while displaying different features, Table 3. In order to select the best pharmacophore model, the features represented by the key residues was considered. Accordingly, a five-featured second model (pharm2) was chosen that has three HBA and two HBD, respectively, as illustrated in Figure 3. The HBAs were found to be represented by the key residues Gln207, Ser228, Lys230, Thr231, while the HBDs have shown interactions with the important residues such as Arg202, Tyr200, respectively, as depicted in Figure 3A and the geometry is shown in Figure 3B.

### 3.2. Validation of the Pharmacophore Models

**Figure 4:**
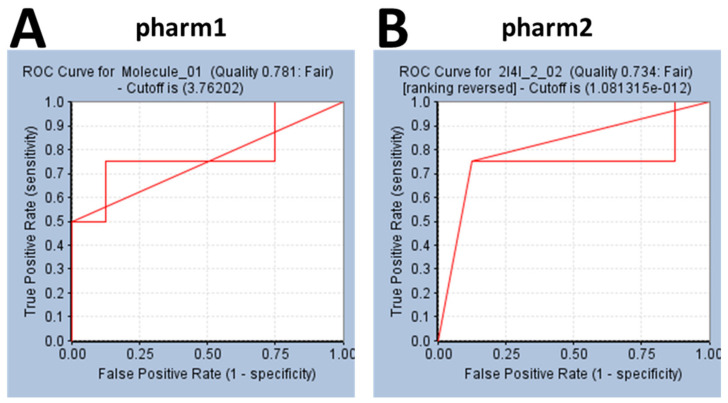
Validation of the pharmacophore model by ROC method. (**A**) Represents the ROC plot of pharm1. (**B**) Indicates the ROC curve of pharm2. Both the models have demonstrated a quality score (greater than 0.7) suitable of employing for virtual screening.

Validation of the pharmacophore models is a crucial step in determining the efficiency of the pharm1 and pharm2 in retrieving the active compounds from a given set of compounds. Accordingly, the ROC was plotted calculating the AUC. Accordingly, the AUC for pharm1 and pharm2 was calculated as 0.78 and 0.73, respectively as shown in Figure 4A,B, proving that the models are capable enough to retrieve the active compounds from the dataset.

### 3.3. Virtual Screening of InterBioScreen Database

**Figure 5:**
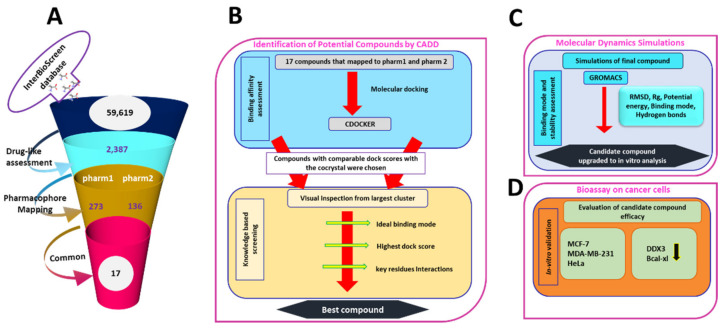
Pictorial representation of workflow to find a prospective compound. (**A**) Interprets the virtual screening method. (**B**) Depicts the molecular docking guided binding affinity studies. (**C**) Illustrates the stability analysis based on molecular dynamics simulation studies. (**D**) Bio assay validation of the selected compound.

The validated pharmacophore models were used as the 3D queries to screen the InterBioScreen database with an objective of obtaining new potential leads. Correspondingly, upon conducting the drug-like assessment, a total of 2387 compounds were yielded that were upgraded and allowed to map with the pharm1 and pharm2 employing the *Ligand Pharmacophore Mapping* tool accessible with the DS retaining all the parameters as default. Subsequently, pharm1 retrieved 273 compounds and pharm2 has mapped to 136 compounds, as depicted in Figure 5A. To obtain the compounds with high inhibitory ability, a manual visual inspection was ventured to select compounds common from both the models. This search resulted in 17 compounds, as illustrated in Supplementary Figure S1. These 17 compounds were forwarded to determine their binding affinities with the protein target DDX3 RNA helicase, 2I4I.

### 3.4. Molecular Docking Studies

Molecular docking is one of the eminent methods employed in the field of molecular biology and computer-assisted drug design [41]. This approach is primarily undertaken to foresee the binding mode(s) of a given small molecules (ligand) with the protein [41] and estimates the binding affinities between them [42]. Additionally, they are used to elucidate the interactions that exists between the ligand and the protein at the atomistic level [41,42]. The 17 compounds acquired from the aforementioned steps were docked into the active site of the protein along with the cocrystal (AMP) as the reference compound. Accordingly, the reference compound has generated a -CDOCKER interaction energy of 50.74 kcal/mol that serves as an upper limit to choose the prospective drug-like compounds, Figure 5B. From the largest cluster and visual inspection for the key residue interactions, one compound has demonstrated the comparable dock score with the cocrystal. This compound was identified as curcumin that has displayed a -CDOCKER interaction energy of 44.08 kcal/mol clamped by several key residues rendered by varied interactions, Figure 5B. Correspondingly, the compound was upgraded to MDS to comprehend on the stability of the system.

### 3.5. Molecular Dynamics Simulation Analysis

MDS approaches are fundamentally employed to decipher on the dynamic molecular motions, functions and to understand the behaviour of protein and ligands at the atomistic level. Correspondingly, 50 ns run was initiated with the best docked conformations as the starting structure and the results were analysed as root mean square deviation (RMSD), radius of gyration (Rg), potential energy, binding mode analysis and hydrogen bond analysis, Figure 5C.

### 3.6. Stability Analysis

**Figure 6:**
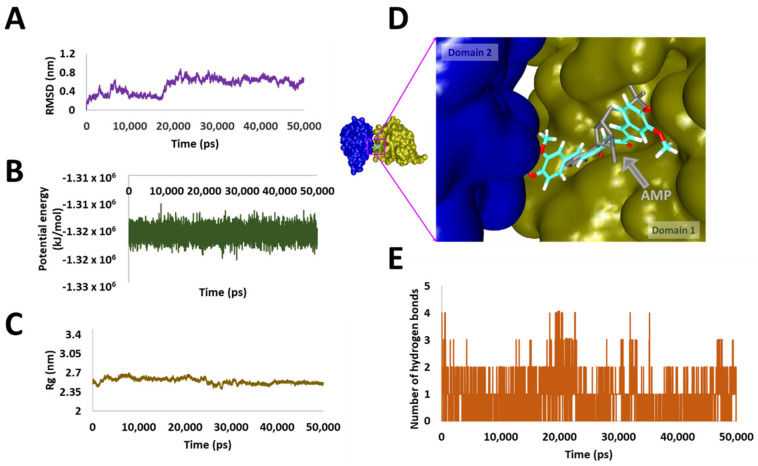
Molecular dynamics simulation analysis. (**A**) Illustrates the root mean square deviation analysis. (**B**) Represents the potential energy profiles. (**C**) Demonstrates the compactness of the protein by radius of gyration. (**D**) Defining the binding mode of the compound at proteins active site. (**E**) Implies the hydrogen bond analysis.

The RMSD analysis has revealed that the system was stability ranged between 0.6 nm to 0.8 nm with an average of 0.5 nm, as illustrated in Figure 6A. Although, a sharp rise in the RMSD was noticed between 16 ns to 18 ns, the system remained stable thereafter. The same was observed from the potential energy profiles as demonstrated in Figure 6B. Additionally, the radius of gyration (Rg) was computed to comprehend on the compactness of the protein, which demonstrated that the protein was compact between 2.35 nm and 2.7 nm as shown in Figure 6C.

### 3.7. Binding Mode Analysis and Intermolecular Interactions

**Figure 7:**
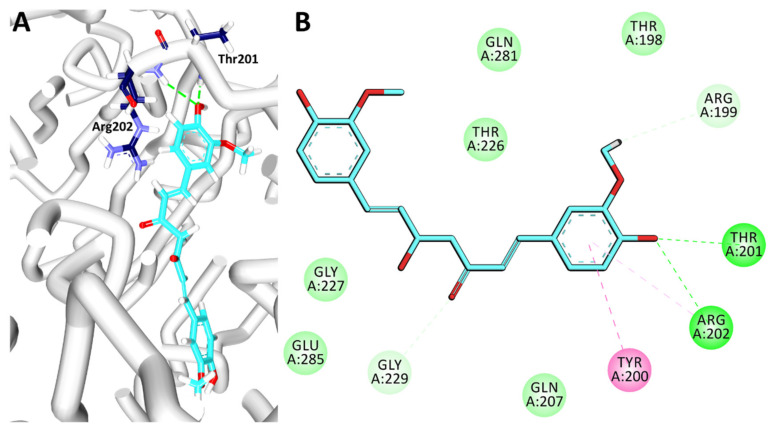
Intermolecular interactions between protein and curcumin. (**A**) Illustrates the hydrogen bond interactions between DDX3 and curcumin. (**B**) Represents the overall intermolecular interactions.

For the binding mode analysis, the representative structures from the last 2 ns were extracted and superimposed against the crystal structure. It was revealed that the curcumin has occupied the binding pocket notably the AMP binding site of domain 1 as depicted in Figure 6D held by several residues. Focusing on the intermolecular interactions, it was detected that curcumin has formed two hydrogen bonds with the residues Thr201 and Arg202 respectively as detailed in Table 4 and Figure 7A. Besides, the residue Tyr200 was observed to form a π-π stacked interaction, while the residues Thr198, Gln207, Thr226, Gly227, Gly229, Gln281 and Glu285 have formed van der Waals interactions assisting to hold the ligand firmly at the active site. The residue Arg202 has additionally interacted with curcumin via π-alkyl interaction. Moreover, the O atom of the residue Arg199 has prompted a carbon-hydrogen bond interaction with the H44 atom of curcumin characterized by a bond length of 3.0 Å. Another carbon hydrogen bond was noticed between the HA1 atom of Gly229 and the O6 atom of the ligand by a bond length of 2.5 Å as illustrated in Figure 7B. Correspondingly, upon monitoring the hydrogen bond interactions, it was noted that the hydrogen bonds were prevalent throughout the simulations as in Figure 6E, illuminating the use of curcumin as DDX3 inhibitor. The compound was evaluated for in vitro analysis as shown in Figure 5D.

### 3.8. Bioassay Validation of Curcumin as Potential DDX3 Inhibitor

#### 3.8.1. Anti-Proliferative Effect and Inhibition of DDX3 Protein Expression of Curcumin

**Figure 8:**
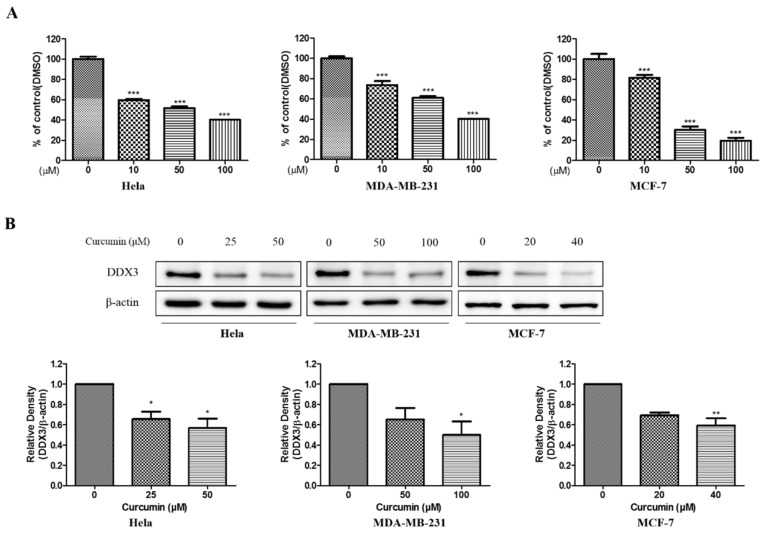
The cytotoxic effects and DDX3 protein expression on curcumin in HeLa, MDA-MB-231, and MCF-7 cell lines. (**A**) The cells were treated with the curcumin as various concentrations (0–100 μM) for 24 h. Then, the cell viability was measured by MTT assay. (**B**) The curcumin was treated with indicated concentration each cell lines for 24 h. Western blot analysis were conducted to identify the DDX3 protein expression. The β-actin protein was used as loading control. * *p* < 0.05 vs. untreated group; ** *p* < 0.01 vs. untreated group; *** *p* < 0.001 vs. untreated group.

To evaluate the anti-proliferative effect of curcumin, MTT assays were carried out on the HeLa cell line, MDA-MB-231 and MCF-7 representing the cervical cancer and human breast cancer cell lines treated with various concentration of curcumin for 24 h. The viability of human cervix and breast cancer cell lines was markedly decreased by curcumin in a dose-dependent manner. The IC_50_ values were recorded as 55.39 μM, 75.46 μM and 31.62 μM for HeLa, MDA-MB-231 and MCF-7 cell line, respectively, as represented in Figure 8A. Based on those results, further experiments were conducted with each concentration curcumin. The three cell lines, which include HeLa, MDA-MB-231 and MCF-7 cells were treated with 10 μM, 50 μM, and 100 μM. The DDX3 protein expression was decreased dose-dependently in three cell lines treated with curcumin as illustrated in Figure 8B This data indicated that curcumin could inhibit DDX3 protein expression in HeLa, MDA-MB-231 and MCF-7 cells.

#### 3.8.2. Anti-Proliferative Effect and Inhibition of DDX3 Protein Level on Exemestane

**Figure 9:**
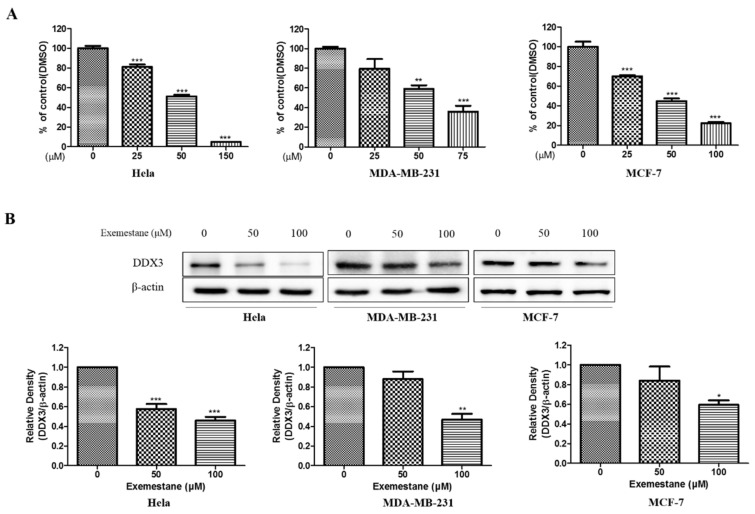
The cytotoxic effects and DDX3 protein expression on exemestane in HeLa, MDA-MB-231, and MCF-7 cell lines. (**A**) The exemestane was treated with indicated concentrations in each cell lines for 24 h. Then, the cell viability was measured by MTT assay. (**B**) The cell lines were treated to each indicated concentration for 24 h. The DDX3 expression level were analyzed using Western blot. The β-actin protein was used as loading control. * *p* < 0.05 vs. untreated group; ** *p* < 0.01 vs. untreated group; *** *p* < 0.001 vs. untreated group.

To examine the cell viability of exemestane, MTT assays were performed in HeLa, MDA-MB-231 and MCF-7 cells treated with various concentrations of exemestane. As shown Figure 9A, the viability was reduced by exemestane in a dose-dependent manner, however at a higher concentration than curcumin. The IC_50_ values is 67.57 μM, 59.29 μM, and 51.30 μM in HeLa, MDA-MB-231 and MCF-7 cell line, respectively, as illustrated in Figure 9A. Correspondingly, further experiments were examined with 50 and 100 μM concentration of exemestane. We also identified the protein level of DDX3 using western blot analysis. As shown Figure 9B, DDX3 protein expression was reduced in exemestane treated three human cancer cell lines. Those date showed that exemestane could decrease DDX3 protein expression in HeLa, MDA-MB-231 and MCF-7 cells.

### 3.9. Exemestane is Synergistic with Curcumin

**Figure 10:**
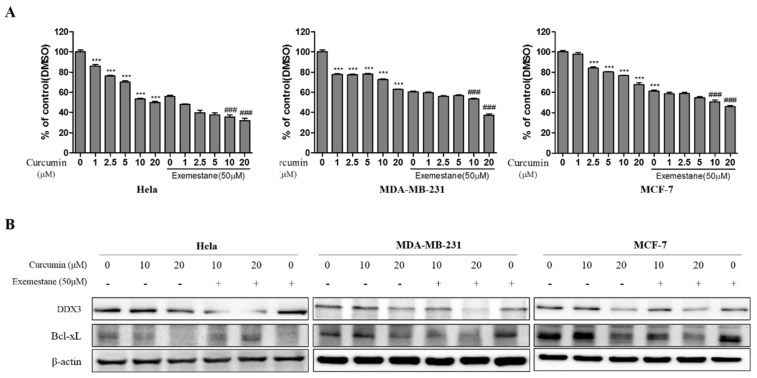
The synergic effects and protein expression of DDX3 and Bcl-xL on combination treatment of curcumin and exemestane in HeLa, MDA-MB-231, and MCF-7 cell lines. (**A**) The various concentration of curcumin with or without 50 μM of exemestane was treated in the cell lines for 24 h. Control group (0 μM) was treated with same amount of DMSO. Then, the cell viability was measured by MTT assay. (**B**) The cell lines were treated with curcumin (10 and 20 μM) with or without exemestane (50 μM) for 24 h. The DDX3 and Bcl-xL expression levels were analyzed using Western blot. The β-actin protein was used as loading control. *** *p* < 0.001 vs. untreated group; ### *p* < 0.001 vs. only the same concentration of curcumin group.

To determine the synergistic inhibitory effect on cell viability, several combination of curcumin (1–20 μM) and exemestane (50 μM; based on IC_50_ value) were examined by MTT assay. The cell viability of combination treated group was more significantly decreased compared with only curcumin treated group in HeLa, MDA-MB-231 and MCF-7 cells as illustrated in Figure 10A. Based on those results, we decide the curcumin (10 and 20 μM) and exemestane (50 μM) for further experiment. In addition, we examined the DDX3 protein expression in co-treated group. As shown Figure 10B, DDX3 protein expression in co-treated group were reduced compared with only curcumin treated group in HeLa, MDA-MB-231 and MCF-7 cells. These results indicated that co-treated groups was synergistic effect in cell viability and inhibition of DDX3 protein expression. In addition, the expression of Bcl-xl protein, anti-apoptotic protein, was decreased in each group of only curcumin or exemestane treatment. The decrease in the Bcl-xl protein was enhanced in the combinatorial treatment than in the individual treated groups. Those data suggests that both curcumin and exemestane treatment can induce apoptosis, and combination treatment can enhance the apoptosis in the three cell lines.

## 4. Discussion

Breast cancer is the leading cause of death in women globally [43,44]. Generally, breast cancer can be classified unto three subtypes depending on the molecular markers [45,46]. With immense advances in the therapeutic field of breast cancer, the chances of disease free survivors have increased enormously, when diagnosed at an early stage and confined to the primary organ [47]. Upon subsequent metastasis, limits the therapeutics with a decline in the success rate [47]. Under such conditions, identifying a target that is elevated in both primary and metastatic stages could be an ideal strategy to successively combat the disease. Therefore, DDX3 would serve as a perfect target as its expression is linked to primary and metastatic cancer samples [22].

Cervical cancer is a preventable disease noticed in women, that affects more than half a million every year with an estimate of 300,000 deaths globally [48]. Cervical cancer is primarily caused due to the human papillomavirus (HPV) [49]. It is evidenced that 18 HPV genotypes are directly associated with cervical cancer, however, more than one type can possibly exist in pre-invasive and invasive cervical cancer [49]. Although, there exists some association between HPV infection and DDX3, a little is known regarding the underlying molecular mechanism between HPV interactions with DDX3 and is yet to be elucidated in HPV-infected cervical cancer [50].

Several inhibitors for DDX3 have been reported earlier. A group of DDX3 inhibitors called the ring-expanded nucleosides (RENs) which were promising antiviral agents have displayed anticancer activities [16]. One such analogue, the NZ51 has shown to decline cellular motility and the viability of the cell in breast cancer cell lines such as MCF-7 and MDA-MB-231 cells at low range of IC_50_ values [21]. Heerma et al., further concluded from their findings that the inhibition of DDX3 induces global cell cycle progression delay by affecting the cells in all phases [8]. Amongst the DDX3 REN agents, the compound RK-33 has a prospective for medicine [16], rendering its activity towards, Ewing sarcoma [18], lung cancer [14], medullablastoma [51], breast cancer [11] and in colorectal [24] and prostate cancer [19]. A recent study has documented the role of doxorubicin against human DDX3 [52]. Radi et al., have reported the discovery of small molecule human DDX3 inhibitor targeting the RNA binding site as potential HIV-1 inhibitors [53]. 1,3,4-thiadiazole inhibitors with potential antiviral activity targeting DDX3 were reported by Brai et al. [54]. In another investigation, avenanthramide A reduced the ATPase activity of DDX3 to combat colorectal cancer [55].

The chosen cell lines represent breast cancer (MCF-7 and MDA-MB-231) and cervical cancer (HeLa). DDX3 is one of the valuable targets to alleviate a host of cancers using small molecules [14,19], Therefore, in the current investigation, we have performed the pharmacophore based virtual screening methods to obtain a potential candidate and evaluated it in vitro in three cell lines. The computationally identified compound curcumin is known to possess several therapeutic applications [56,57,58,59] besides possessing anticancer properties [60,61]. Owing to the wider therapeutic applications of curcumin, we evaluated curcumin against DDX3. To the best of our knowledge, this is the first report to document the effect of curcumin on DDX3. The in vitro results have demonstrated that curcumin has downregulated the expression of DDX3 in three cell lines including, MCF-7, MDA-MB-231 and HeLa cell lines.

We were also interested in targeting DDX3 with an approved drug and therefore, exemestane was chosen. The compound exemestane is known as aromatase inhibitor for breast cancer [62,63]. Exemestane was docked into the binding site of 2I4I to estimate the binding affinities between the DDX3 and small molecules. Corresponding results have shown that exemestane has occupied the binding site of the protein rendered by a dock score of 22.93 kcal/mol. Several key residues were found to hold the compound firmly at the active site. The HN atom of Gly227 has interacted with O2 atom of exemestane compound by a bond length of 2.2 Å forming a hydrogen bond. The HA atom of the residue Thr226 and O2 atom of exemestane have formed a carbon hydrogen bond interaction with the a bond length of 2.8 Å. The key residue Tyr200 has prompted two π-alkyl interactions with the ligand. The residues Arg202, Thr204, Gln207, Thr226, Gly229, Lys230, Thr231, Ala232 and His527 have aided in accommodating the compound at the active site of the protein via the van der waals interactions. There findings have encouraged us to escalate it to in vitro assay. Upon treating the three cell lines with exemestane, the expression of DDX3 was reduced, as shown in Figure 9. This triggered our interest to perform the combination study of curcumin and exemestane and estimate the DDX3 expression. The results have indicated that DDX3 expression was reduced significantly upon combinatorial treatment than single/individual compound.

The compound curcumin has formed two hydrogen bond interactions with the key residues Thr201 and Arg202 as reported earlier [2,52]. The residue Arg202 is recorded to be a key residue originating from the Q motif of the protein binding pocket of the DDX3 as noticed in the crystal structure. Our findings also highlighted this crucial interaction, proclaiming that curcumin could be employed against DDX3.Taken together, our findings proclaim that curcumin could act has an individual drug or could be a better option with exemestane to treat DDX3 elevated cancers. Furthermore, our findings pave the way for new research avenues and serves as a fundamental platform for combinatorial treatments.

## 5. Conclusions

The current study aims at identifying a potential candidate to target DDX3. DDX3 has been implicated in several cancers and targeting DDX3 could be an ideal strategy to abrogate cancers. In this pursuit, several in silico investigations were executed to identify potential compound and further validated in vitro. The computational approaches have identified curcumin as a prospective candidate, which had reduced the expression of DDX3 in three cell lines. However, upon administering curcumin along with exemestane, the expression of DDX3 was reduced remarkably. These findings could pave the way for new research avenues and may assist in designing potential candidate for DDX3. 

## Figures and Tables

**Table 1:** List of inactive compounds considered for pharmacophore validation.

Compound No.	SMILES	IC_50_ nM	ChEMBL ID
Compound **1**	Oc1cccc(NC(=O)CCN2C(=S)S\C(=C/c3cccc(Br)c3)\C2=O)c1	90,000	CHEMBL457233
Compound **2**	Oc1cccc(NC(=O)CCCN2C(=S)S\C(=C/c3cccc(Br)c3)\C2=O)c1	150,000	CHEMBL456405
Compound **3**	COc1ccccc1\C=C\2/SC(=S)N(CCC(=O)Nc3cccc(O)c3)C2=O	200,000	CHEMBL514760
Compound **4**	OC(=O)c1ccccc1NC(=O)CCN2C(=S)S\C(=C/c3cccc(Br)c3)\C2=O	200,000	CHEMBL484400
Compound **5**	Nc1c(C#N)c(nn1c2ccccc2)\C(=C\c3oc(cc3)c4ccccc4[N+](=O)[O-])\C#N	300,000	CHEMBL484749
Compound **6**	Oc1ccc(NC(=O)CCN2C(=S)S\C(=C/c3cccc(Br)c3)\C2=O)cc1	300,000	CHEMBL515669
Compound **7**	Nc1c(C#N)c(nn1c2ccccc2)\C(=C\c3oc(cc3)c4cccc(Cl)c4)\C#N	500,000	CHEMBL500746
Compound **8**	Oc1ccccc1NC(=O)CCCN2C(=S)S\C(=C/c3cccc(Br)c3)\C2=O	500,000	CHEMBL459179

**Table 2:** Generation of different pharmacophore models and their corresponding features.

Model No.	Features *	Rank	Direct Hit	Partial Hit	Max Fit
01	2HA,HYP,HBD	31.233	1111	0000	4
02	HA,HYP,HBD,RA	30.714	1111	0000	4
03	HA,HYP,HBD,RA	30.629	1111	0000	4
04	HA,2HYP,HBD	28.033	1111	0000	4
05	HA,2HYP,HBD	28.033	1111	0000	4
06	2HA,HBD	27.918	1111	0000	3
07	2HYP,HBD,RA	27.514	1111	0000	4
08	HA,HBD,RA	27.470	1111	0000	3
09	HA,HBD,RA	27.438	1111	0000	3
10	2HYP,HBD,RA	27.429	1111	0000	4

* Hydrogen bond acceptor (HBA), hydrogen bond donor (HBD), hydrophobic (HYP), hydrophobic aromatic (HA) and ring aromatic (RA).

**Table 3:** Varied pharmacophore models and features derived during the receptor pharmacophore generation.

Model No.	Number of Features	Feature Set *
01	5	HBA,HBA,HBD,HBD,RA
02	5	HBA,HBA,HBA,HBD,HBD
03	5	HBA,HBA,HBD,HBD,RA
04	5	HBA,HBA,HBA,HBD,HBD
05	5	HBA,HBA,HBD,HBD,RA
06	5	HBA,HBA,HBD,HBD,RA
07	5	HBA,HBA,HBD,HBD,RA
08	5	HBA,HBA,HBD,HBD,RA
09	5	HBA,HBA,HBD,HBD,RA
10	5	HBA,HBA,HBD,HBD,RA

* Hydrogen bond acceptor (HBA), hydrogen bond donor (HBD), and ring aromatic (RA). All the models have generated a selectivity score of 9.9146.

**Table 4:** Intermolecular interactions observed between DDX3 and curcumin.

Compound	Hydrogen Bonds	π-π Stacked	van der Waals Interactions
Curcumin	Thr201: HN-O3 (2.2 Å)	Tyr200	Thr198, Gln207, Thr226, Gly227, Gly229, Gln281, Glu285
	Arg202: HN-O3 (2.3 Å)		

## Supplementary Materials

The following are available online at https://www.mdpi.com/2218-273X/10/6/857/s1, Figure S1: Alignment of the retrieved compounds to pharmacophore models.
