# Supplementary material for: A Computational Approach with Biological Evaluation: Combinatorial Treatment of Curcumin and Exemestane Synergistically Regulates DDX3 Expression in Cancer Cell Lines
Source: Biomolecules. 2020 Jun 4;10(6):857. doi: 10.3390/biom10060857 (PMC7355417; doi:10.3390/biom10060857)
Supplement: Supplementary file 1 [file biomolecules-10-00857-s001.pdf]

Structure-based

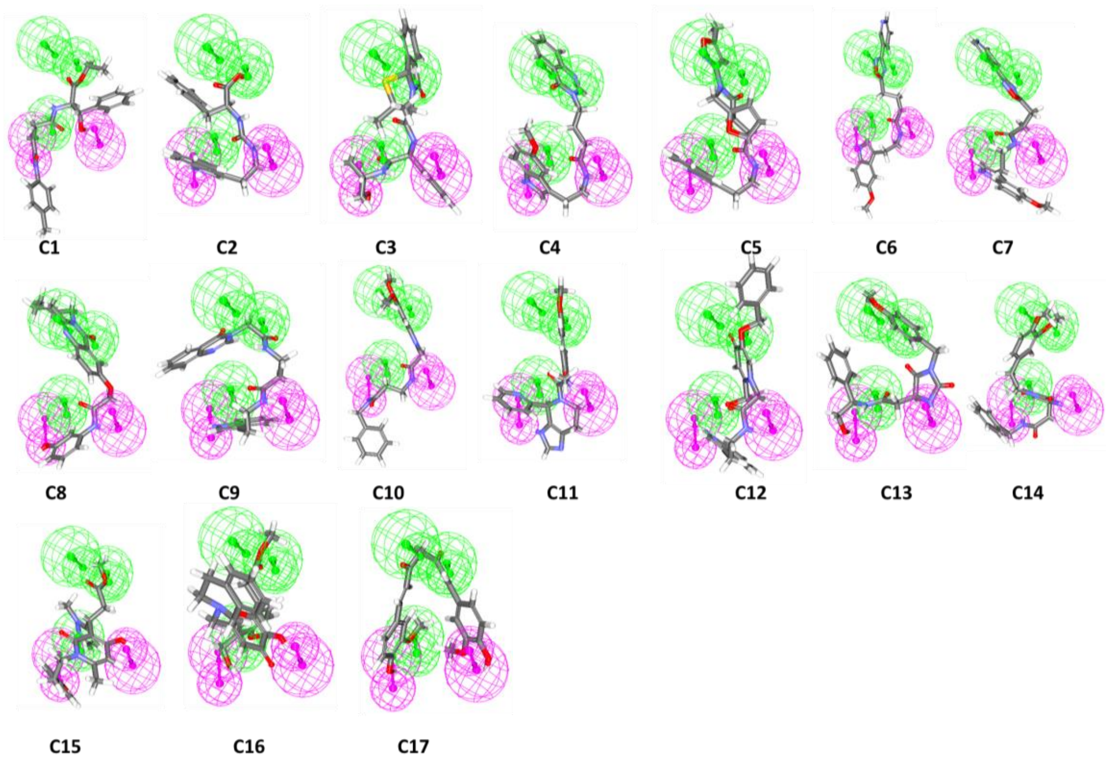

Ligand-based

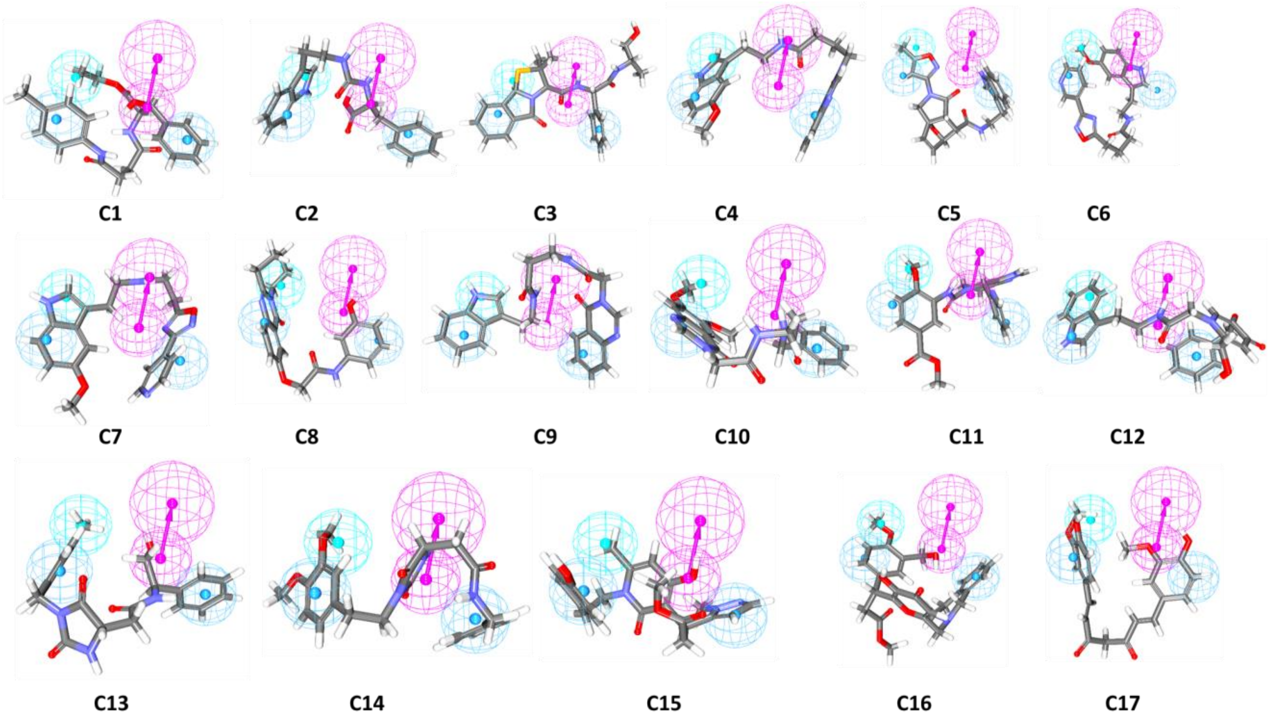

Supplementary Figure 1: Retrieved compounds alignment to pharmacophore models
